# Violating Social Norms when Choosing Friends: How Rule-Breakers Affect Social Networks

**DOI:** 10.1371/journal.pone.0026652

**Published:** 2011-10-21

**Authors:** Karlo Hock, Nina H. Fefferman

**Affiliations:** 1 Department of Ecology, Evolution & Natural Resources, Rutgers, The State University of New Jersey, New Brunswick, New Jersey, United States of America; 2 DIMACS, Rutgers The State University of New Jersey, Piscataway, New Jersey, United States of America; University of Maribor, Slovenia

## Abstract

Social networks rely on basic rules of conduct to yield functioning societies in both human and animal populations. As individuals follow established rules, their behavioral decisions shape the social network and give it structure. Using dynamic, self-organizing social network models we demonstrate that defying conventions in a social system can affect multiple levels of social and organizational success independently. Such actions primarily affect actors' own positions within the network, but individuals can also affect the overall structure of a network even without immediately affecting themselves or others. These results indicate that defying the established social norms can help individuals to change the properties of a social system via seemingly neutral behaviors, highlighting the power of rule-breaking behavior to transform convention-based societies, even before direct impacts on individuals can be measured.

## Introduction

Social interactions determine positions of individuals within their group (and the associated fitness consequences), and also define a quantifiable social structure indicative of the overall group organization [1]–[7]. While relative success of behavioral strategies in a population will depend on their selective (dis)advantages [8]–[10], breaking the rules of conduct will inevitably affect others [11]–[14]. Defying social norms has the potential to change the functioning of an entire system [15]–[17], affecting the social success and/or fitness of all group members. For example, an individual's actions could facilitate population-wide change by making other strategies less viable through mechanisms such as selection or cultural transmission [8], [18], as well as co-evolution in social dilemmas and similar evolutionary games based on cooperation [7], [9], [19], [20], reviewed in [10]. Similarly, the interaction structure of a group may be altered by behavioral actions of individuals, disrupting processes such as flow of information [21] or connectedness of different network components [22], [23]. Group organization may be important if it helps the emergence of key individuals which will then have a beneficial effect on survival of the entire group [22], [24]. Removal or disruption of the emergence of such individuals may therefore harm all involved, even though immediate social positions of other individuals are not directly altered [16], [25], [26]. A system's social structure could also be more or less robust to the effects of behavioral decisions that break the rules of established norms [27]. If living within a social network with clearly defined structure and rules of conduct has benefits, for example by reducing the need for repeated tests of competence from interacting individuals [28], rule-breaking behavior has the potential to transform the system beyond its immediate impacts, altering not only the expected social success at the individual level, but also the structure and global properties of a system. The consequences of such rule violations should therefore be investigated not only through ramifications for the individuals that perform them, but also through the broader social impacts of rule-breaking behavior on network properties.

Elegant studies have already investigated how individual cooperative behaviors, motivated by self-interest and returns from cooperative strategies, can be selectively maintained from the perspective of evolutionary game theory [7], [9], [10], [19], [20]. Further work has explored the emergence of institutional organizations from these strategies [29]–[31], frequently relying on reinforcement from either reward [32]–[34] or punishment [17], [35]–[38]. To complement and contrast these game-theoretic methods, in which rational individuals choose social partners or strategies on the basis of known fitness payoffs from games, we instead focused on a system in which individuals can evaluate only relative network positions of partners as indirect proxy measures for potential payoffs, but are unable to accurately predict the fitness impacts of their choices. To study the emergence of organization and potential for individual and organizational success in such groups, we therefore simulated a network of individuals that chose their social partners exclusively by using partner's perceived social prominence. Such a framework is particularly well suited for this purpose [39], as social networks permit quantification of both individuals' personal positions within a group [6], [40] and the global properties of an entire group [41], [42]. Our networks featured dynamic, individual-based behavioral rules, in which each individual's social prominence fluctuated as a result of social decisions of others, while system-wide properties emerged from this self-organization. In these simulations, all social choices were free: individuals were only concerned with retaining desirable social partners and did not consider the effects of their actions on others nor did they coordinate actions to achieve a common good. Systems of this kind yield stable emergent properties of social structure despite their inherently stochastic nature [39], allowing independent characterization of the impacts of divergent behavioral decisions at multiple levels.

**Figure 1:**
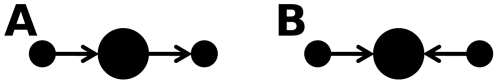
A 3-node network examples demonstrating how individual centrality metrics were measured. A) Betweenness centrality: individual in the middle is the necessary intermediary between the left and right individual, as it lies on the shortest path between those individuals; the middle individual therefore has higher individual betweenness centrality than the other two individuals; B) In-degree centrality: both left and right individual have connections towards the middle individual, making the middle individual ‘popular’ as a partner; middle individual therefore has higher individual in-degree centrality than the other two individuals.

Individuals in our networks chose ‘friends’ using two different measures of social centrality (Fig. 1; see also [6], [40], [43]), either according to the partner's quality as a necessary intermediary between others (also called betweenness) or according to the partner's popularity (also called in-degree), to determine which affiliations to maintain. While these measures may be considered proxies for any evaluative metric by which a self-organizing social system yields differences in centrality among individuals [6], populations that employ such centrality measures or their proxies, and their potential implications for individual fitness, have been documented in real-world networks [13], [14], [22]. Individuals that did not follow these rules of conduct instead changed their social partners at random, thus breaking the social conventions used by the rest of the group. Initially, all affiliations were assigned randomly and all individuals had the same probability of being chosen as partners. This random initial structure then dictated each individual's desirability as a partner according to the assigned affiliation criteria: for individuals that followed conventions, the more prominent the individual's social position, the more likely others were to remain affiliated with it. The criteria for affiliation were treated as simple social conventions [44], [45]: while they may or may not have specific fitness analogues in particular systems, in our simulations they served only to drive the self-organizing behaviors in non-deterministic social systems.

**Figure 2:**
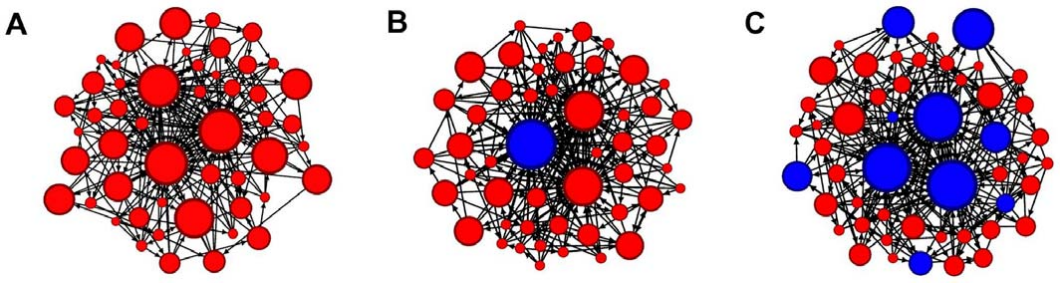
Examples of intermediary-based networks. A) Uniform intermediary-based network; B) Intermediary-based network with a single rule-breaking individual; C) Intermediary-based network with 20% of individuals that did not follow this convention. While the simulations were not spatially explicit, the size of the individuals in a network is proportional to its quality as an intermediary (betweenness centrality). Individuals that broke the rules of conduct (identified by their blue color) enjoyed progressively higher social success as they increased in frequency, whereas the success of the convention-abiding individuals decreased at the same time.

**Figure 3:**
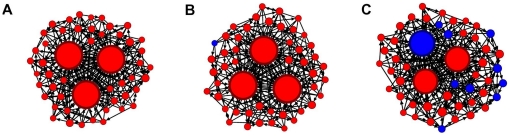
Examples of popularity-based networks. A) Uniform popularity network; B) Popularity network with a single rule-breaking individual; C) Popularity network with 20% of individuals that did not follow this convention. While the simulations were not spatially explicit, the size of the individuals in a network is proportional to that individual's popularity (in-degree centrality). Even though the individuals that used rule-breaking behavior (identified by their blue color) had social success comparable to the convention-abiding individuals, the emergent properties of the system changed: with more individuals not playing by the rules, the group became more decentralized.

To determine how breaking the rules of conduct affects a social network, we compared networks of individuals who all preferred partners with high individual centrality (by either the quality-as-intermediary or popularity metric; Figs. 2A and 3A, respectively) to those featuring different frequencies of rule-breakers: either with only a single rule-breaking individual (Figs. 2B and 3B) or with a substantial proportion (20%) of rule-breaking individuals (Figs. 2C and 3C). We then calculated the individual centrality that each individual could expect to attain, and the group-wide level of centrality as an emergent property of the group. By calculating the impact of rule-breaking behavior at different levels of organizational success, we determined that it is possible to affect the organizational structure of a group without directly affecting the social positions of individuals. Such system-level consequences offer quantitative insights into the transformative powers of rule-breaking behavior in groups that adhere to social norms and conventions and/or rely on the network structure to function effectively.

## Results and Discussion

Behavioral decisions by which individuals chose their social partners significantly affected both their own position and the social organization of the group. Moreover, the two scenarios produced markedly different results: individuals primarily affected their own social position in the intermediary-based networks, while in the popularity-based networks they affected the overall social structure without producing any immediate effects at the individual level.

**Figure 4:**
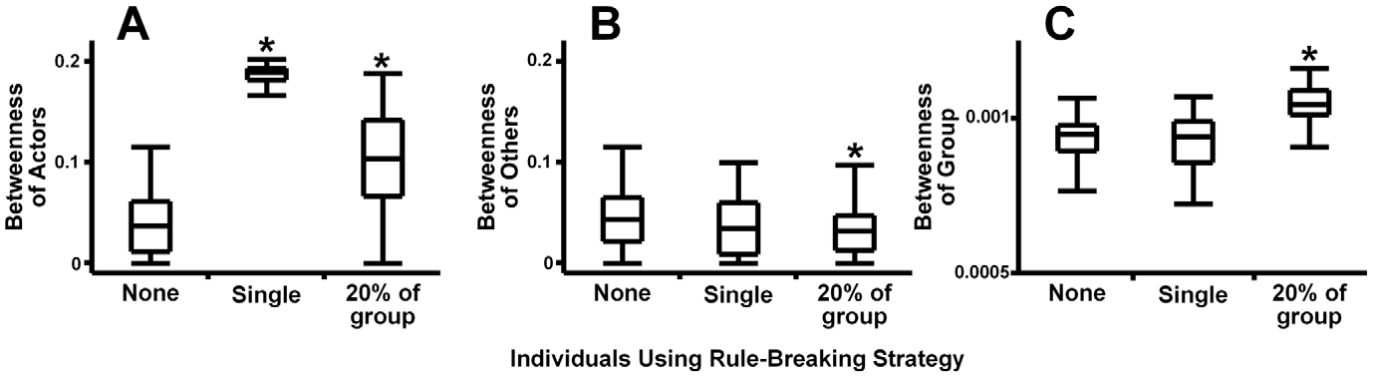
Effects of rule-breakers on intermediary-based networks. Individuals using rule-breaking strategy primarily affected (A) their own social position (H(2) = 213.18, p<0.0001), but also made an impact on the other aspects of the social system, affecting (B) the social position of others (H(2) = 6.79, p = 0.034) and (C) group organization (H(2) = 125.3, p<0.0001) as their frequency in a population increased. The boxes show medians, quartiles, minima and maxima. Results significantly different from a relevant uniform network with no rule-breaking behavior (p<0.05 in Dunn's multiple comparison test for comparing each group with control) are designated with *.

When intermediary quality was the social convention guiding partner choice, individuals that broke the social rules of conduct affected their own success (Fig. 4A), the social success of convention-abiding majority (Fig. 4B), and the group organization as a whole (Fig. 4C). Even a single individual that affiliated randomly directly affected its own social position. Such direct consequences would then either cause such rule-breaking behavior to spread in, or disappear from, the population. However, since a single individual had no effect on others and did not affect the group-wide organization, breaking social rules would make it either more or less successful than the rest. This effect alone would then determine whether rule-breaking would become more prevalent. If rule-breaking becomes more common due to a positive effect on that individual, the initial advantage would persist while the success of convention-abiding individuals, as well as group-wide organization, would change. Thus, in the intermediary examples, breaking conventions primarily affected the individual itself, and only influenced other levels of a social system at higher frequency. Sufficient punishment (such as fitness loss from not connecting to successful intermediaries) would therefore prevent a single individual from changing the success of a group, whereas a reward to the rule-breaking individual could eventually bring down the entire system. This is consistent with observed mechanisms in animal and human societies that prevent rule-breaking, maintaining the functional structure of a group [46], [47].

**Figure 5:**
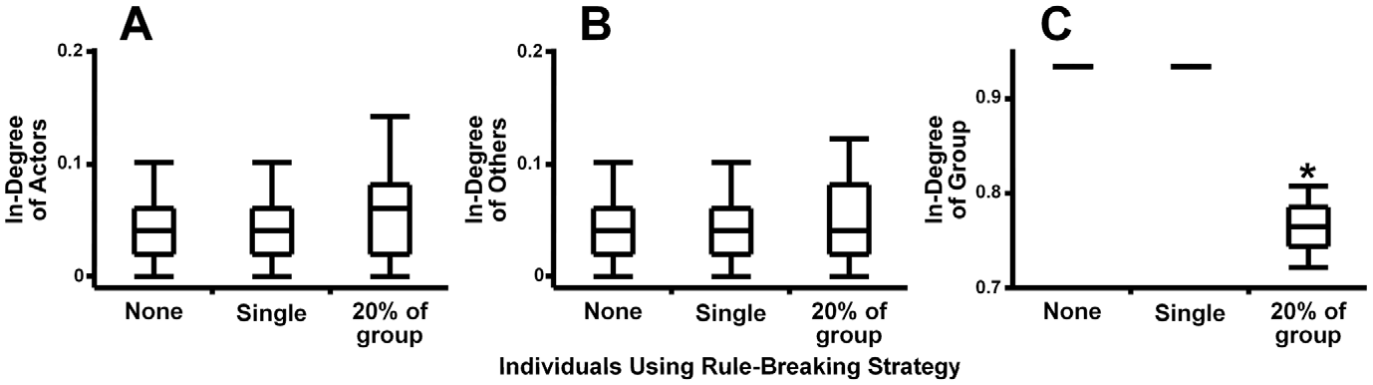
Effects of rule-breakers on popularity-based networks. Individuals using rule-breaking strategy did not significantly affect either (A) their own social position (H(2) = 2.99, p = 0.22) or (B) the social position of others (H(2) = 0.24, p = 0.89). However, as their presence in the population increased, the behavior of these individuals will start to make an impact, affecting (C) the overall group organization (H(2) = 284.55, p<0.0001) even though there is no concurrent measurable effect on the individual values. The boxes show medians, quartiles, minima and maxima. Results significantly different from a relevant uniform network with no rule-breaking behavior (p<0.05 in Dunn's multiple comparison test for comparing each group with control) are designated with *.

Interestingly, in popularity-based networks rule-breaking individuals did not affect their own success (Fig. 5A) or the social success of others (Fig. 5B), but they did affect the overall group organization (Fig. 5C). As a single individual was not able to produce this effect, breaking the social rules of conduct would initially appear to be completely neutral to the social network and its constituents. Rule-breaking individuals would have the same success as if they abided by conventions, and the convention-abiding majority would not be affected. However, if rule-breaking behavior was then to increase in frequency (for example, due to chance in the absence of strong pressure either for or against it), the success of either rule-breaking or convention-abiding individuals themselves would still not change, but the structure of the group as a whole would. In these popularity-based networks, rule-breaking individuals were able to change the structure of a social network and the nature of how an entire social system works, achieving this without necessarily affecting any individual in particular. Despite their potentially global impact, it was only possible to gauge the true influence of these individuals on the network by taking into account the system-wide properties. If a specific network structure is essential for the community (for example, in hierarchies or other highly centralized network or organization where most individuals connect only to the few most popular individuals) a growing number of rule-breakers or dissenters could affect the success of the entire social group [16], [29].

These multifaceted effects on organized social systems demonstrate that even simple changes in social behavior can affect systemic social network properties, which in turn challenges the way we think about animal and human social networks. Though the level at which we observe the costs and benefits must be an individual, the full extent of the impact from individuals' actions cannot be gauged without exploring their effects on a community or a population as a whole. Many social systems, both animal and human, are based on social rules of conduct which are not always (evolutionarily or socially) optimal (such as certain sexual taboos [46], or respect for resource ownership [48]), but which are also not always arbitrary [48], [49], and frequently help make the system more efficient [26], [29], [37], [47], [50], [51]. Whereas violation of social rules often carries direct consequences for the actor, such as through direct punishment or withdrawal of future cooperation [17], [35], [37], [38], [52], [53], even behavioral decisions that seem immediately neutral for the involved individuals (and likely would not provoke a punishment) can still profoundly change how the system works. The studies of such actions should therefore consider system-wide properties of both animal and human social networks without sacrificing any attention to the individual-level consequences of social actions. By changing the properties of the network on a global level, a single individual has the potential to change its entire social environment by the power of its own actions alone. We should therefore aim to determine such systemic consequences of social behaviors and rule-breaking decisions before any such actions can be described, and dismissed, as truly neutral.

## Materials and Methods

### Modeling environment and dynamics

The modeling environment was designed as a graph *G*, in which *n* individuals were represented as nodes, or vertices, *V* = {*v_1_*, *v_2_*, …, *v_n_*} in a network and their interactions as connections between those nodes. Interactions were directed, in that the distinction was made between sources and recipients of social interactions so that an individual *v_i_*'s choice to be affiliated to another individual *v_j_* was represented by an arc (*v_i_, v_j_*) in *G*, thus resulting in a directed graph, or digraph. If an arc (*v_i_, v_j_*) exists, individual *v_j_* was said to be an out-neighbor of individual *v_i_*. These connections were then used to determine centrality metrics of individual nodes (individual centrality), measures of position and connectivity in an interaction network, as well as centrality of the digraph as a whole (group-wide centrality). This digraph structure was then used as a basis for development of a dynamic, self-organizing, individual-based social network framework in which individuals could display different social affiliation preferences. All affiliations between individuals were freely formed, and individuals could only directly affect the interactions they themselves initiated (that is, choose their own out-neighbors), but not the actions of other individuals (they could not directly affect the choices of others to select them as out-neighbors or not). Each network consisted of a constant number of 50 individuals in total, with each individual assigning outgoing connections to five out-neighbors. Individuals had no inherent personal advantages that would prime them to be selected as social partners, and all subsequent choices were made using dynamic centrality properties to assess out-neighbors. When the network was initialized the connections between all individuals were assigned randomly, giving a graph *G_0_*, but were then updated according to the built-in preference of the individuals for partners of certain centrality.

The ability of individuals to choose their ‘friends’ by dropping the connections to existing out-neighbors and forming connections to new out-neighbors resulted in a self-organizing dynamic network in which the organization of connections was constantly updated in accordance to the preset affiliation rules. The dynamics of the models took place in discrete time steps. At each time step *t* in graph *G_t_*, each individual ranked the centrality values of its five out-neighbors according to its predetermined affiliation preference. It then opted to remain affiliated with out-neighbors it perceived as desirable social partners by retaining the 3 highest ranking ones while dropping the 2 lowest ranking ones. It then added 2 new out-neighbors at random from the entire group (excluding the two it just dropped) before next time step, which completed the iteration and (when completed by all individuals) resulted in graph *G_t+1_*. Alternatively, individuals could also drop out-neighbors completely at random, thus foregoing the conventional assessment of out-neighbor quality through centrality comparisons in their affiliation choices. Such individuals were denoted as exhibiting divergent strategy, abandoning the conventional choice of social partners and using an alternative, rule-breaking strategy in an otherwise convention-abiding, ordered group of individuals. In all scenarios, the total edge density of the network was constant since, in each *G*_t_, each individual always had exactly five out-neighbors; the only parameter that varied between iterations was the arrangement of connections.

Our goal was to demonstrate the impact of rule-breaking on an organized group of individuals, rather than achieve any optimization of social organization in a group. As such, the individuals did not coordinate their actions to achieve a common goal, did not consider the effects of their actions on others, and in general did not behave optimally in an evolutionary sense as there are likely to be many behaviors, and/or centrality measures by which to evaluate them, that could do a better job at maximizing individual's social prominence [39], such as coercion or cheating. We also did not aim to ascribe any qualitative importance to centrality in a social network as a measure of prominence in a biological group; specific selection pressures can cause either high or low betweenness or in-degree centrality to be considered positive [13], [14], [22] or even to have no directly measurable fitness correlates. We instead used these very simple social conventions to illustrate the fact that even the simplest affiliation differences can result in distinct and quantifiable changes in group organization properties even with constant density of social contacts, and that such properties can be disrupted by breaking the rules of social conduct.

### Centrality measures for individuals and groups

As neither the rules of affiliation nor initial characteristics of individuals inherently implied any position for individuals or social structure of a group, centrality measures for individuals and groups were emergent properties of the social structuring processes. Two centrality measures were used as proxies of social quality of partners [6], [39], [40], [54]: betweenness and in-degree. Betweenness *B* of individual *v_i_* is defined as
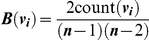



where count(*v_i_*) is the number of shortest paths between any two individuals in a network that contain node *v_i_* as an intermediate node, and *n* is the total number of individuals in a network. Betweenness measures how essential an individual is as a necessary intermediary between pairs of individuals. In-degree *D* of individual *v_i_* is defined as
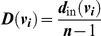



where *d_in_*(*v_i_*) is the number of individuals that form connection to *v_i_*, and *n* is the total number of individuals in a network. It is essentially equal to the number of incoming connections to *v_i_*, measuring how ‘popular’ *v_i_* is as a partner. In addition to measuring centrality of individuals, centrality of groups as an emergent property was also quantified. Group-wide betweenness *B* of group *G* is measured as
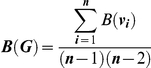



where *B*(*v_i_*) denotes the betweenness of individual *i*, and *n* is the total number of individuals in a network. Group-wide in-degree *D* of group *G* was measured as
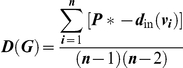



where *d*_in_(*v_i_*) is the number of individuals that form connection to *v_i_*, P* = max [*d_in_*(*v_i_*) | *i* = 1,…,*n*], and *n* is the total number of individuals in a network. Both of these group-wide metrics quantify how well the group is organized as a whole according to the respective centrality measure of individuals.

### Simulations and analyses

There were a total of 6 network types: 3 betweenness-based (a uniform betweenness network consisting of only betweenness individuals, a betweenness network with a single rule-breaker affiliating randomly, and a betweenness network with 10 randomly affiliating rule-breakers), and 3 in-degree based types (a uniform in-degree network consisting of only in-degree individuals, an in-degree network with a single rule-breaker affiliating randomly, and an in-degree network with 10 randomly affiliating rule-breakers). We ran 100 independent Monte Carlo realizations per network type, and recorded the respective centrality measures of each individual and group centrality after 200 time steps. Both numbers were deemed statistically sufficient due to convergence of outcome variance and comparable stability calculations, as described in [54]. We used networks in which all individuals had an identical preference, preferring to remain affiliated with partners of either high betweenness or high in-degree, as a basal state against which subsequent scenarios were compared. A random subset of the sampled measures was used in all comparisons so as to account for differences in sample sizes, giving the sample size of 100 for each group in each comparison. We used nonparametric tests (two-way Kruskal-Wallis ANOVA and corresponding nonparametric Dunn's post-hoc comparisons of each group with control) in statistical analyses due to the heteroscedasticity and non-normal distributions of the results. The networks were visualized using Gephi 0.8 (Bastian M, Heymann S, Jacomy M, unpublished).
